# Hahnemann’s Tomb

**Published:** 1899-06

**Authors:** Bushrod W. James

**Affiliations:** American Representative of the Commission; N. E. Cor. Eighteenth and Green Sts., Philadelphia, Pa.


### HAHNEMANN'S TOMB.

To the Members of the Homœopathic Medical Profession of the United States:

The Commission appointed at the last Medical Congress in London, for the restoration of Hahnemann's Tomb is actively at work. All who have subscribed to the fund should send in their unpaid subscriptions at once.

Any members of the profession, or laity, desiring to contribute anything further towards this restoration of Hahnemann's Tomb in Père La Chaise Cemetery, at Paris, France, should forward the amount at once, either to me, as the American Representative, or to Dr. François Cartier, the Secretary, at 18 Rue Vignon, Paris. The Commission has thus far collected about fifteen thousand francs, which will be utilized to the best advantage. Thanking all who have contributed, and trusting the work of the Commission will be acceptable to the homœpathic profession of the world, I am,

Fraternally yours,

Dr. Bushrod W. James,

*American Representative of the Commission.*

N. E. Cor. Eighteenth and Green Sts., Philadelphia, Pa.

